# Center of mass direction and speed during a 45-degree change of direction task performed with maximal effort

**DOI:** 10.3389/fspor.2025.1576614

**Published:** 2025-06-05

**Authors:** Daichi Yamashita, Yuki Inaba, Masaki Asakura, Yoshihiko Ito

**Affiliations:** ^1^ Department of Sport Science, Japan Institute of Sports Sciences, Kita, Tokyo, Japan; ^2^ School of Movement, Yokohama, Kanagawa, Japan; ^3^ DEERS Football Club, Tainai, Niigata, Japan; ^4^ R&S Company, Shinagawa, Tokyo, Japan

**Keywords:** cutting, kinetics, agility, multi-step mechanism, acceleration, deceleration

## Abstract

Changes in whole-body center of mass (COM) direction and speed over multiple steps during a maximal effort change of direction (COD) task have not been fully examined. This study aimed to (1) quantify COM direction and speed changes across three steps —approach (APP), execution (EXE), and following (FOL)—during a 45° COD task, and (2) compare force production between EXE and FOL. Ten male American football players performed straight running (RUN) and sidestep cutting to a 45° COD (COD45) tasks. In RUN, participants sprinted 15 yards (13.73 m) at maximal speed. In COD45, they sprinted 10 yards (9.15 m), executed a 45° cut, and completed an additional 5-yard (4.58 m) sprint. COM speed and direction were analyzed across four flight phases in COD45 (FLIGHT-2, FLIGHT-1, FLIGHT + 1, FLIGHT + 2) and three in RUN (FLIGHT-2, FLIGHT-1, FLIGHT + 1). Horizontal ground reaction impulses (GRIs) during EXE in RUN and EXE and FOL steps in COD45 were analyzed in a local coordinate system aligned with the COM velocity vector. Although COM speed remained unchanged during EXE (between FLIGHT-1 and FLIGHT + 1; *p* = .053), this step produced a greater medial GRI than FOL (*p* < .001); however, the direction change during this step was only 15.30°, one-third of the required 45°. APP and FOL contributed 9.70° and 9.05°, respectively, to the direction change while simultaneously increasing COM speed by 0.23 m/s and 0.13 m/s, respectively. Therefore, completing a maximal effort 45° COD requires multi-step role sharing, incorporating both directional changes and acceleration.

## 1 Introduction

The ability to change direction is essential in competitive sports such as American football, basketball, and soccer (1–4). To gain separation from an opponent, athletes need to run fast and perform changes of direction quickly and with maximum effort. Athletes competing at a higher level in invasion sports such as soccer, rugby union, and American football have shorter completion times than those at a lower competitive level (5–7).

Previous studies have investigated the biomechanical characteristics of a single foot, referred to as the “execution step” (EXE) (8), or the “plant step” (9), during a change of direction (COD) task. Havens and Sigward (10) reported that the EXE produces great braking and medial ground reaction impulses (GRIs), which contribute to decelerating and rotating the whole-body center of mass (COM) velocity vector during a 45° COD task performed with maximal effort. They also showed that the approach step (APP; one step before EXE) produces greater braking and medial GRI and changes the COM direction compared with straight sprinting (10).

Moreover, Vanrenterghem et al. (11) reported that after the EXE takeoff, the COM velocity vector does not achieve the required direction during a 45° COD task, even at submaximal speeds (2–5 m/s), and the directional change during the EXE is smaller with increasing running speed. Some studies have shown that COD tasks are completed with multi-step sharing roles (9, 12), at running speeds (13) and even at walking speeds (i.e., activities of daily living) (14, 15). Although the great contribution of the following step (FOL; one step after EXE) performed during maximal effort COD can be easily expected, its contribution remains unknown.

In experimental settings that include COD tasks with submaximal speeds, previous studies reported that the APP and EXE play roles in decelerating the COM speed (8, 11, 16). However, in sports, COD tasks are usually conducted during the acceleration phase to evade or catch an opponent. Hader et al. (2) and Andrews et al. (17) stated that there are three phases of COD tasks: acceleration, deceleration, and re-acceleration. Nevertheless, no study has quantified the change in COM direction and speed that occurs with multiple steps during a COD task that is performed with maximal effort (e.g., when and how an athlete accelerates, decelerates, and re-accelerates while changing the COM direction).

Therefore, this study aimed to (1) quantify COM direction and speed changes over three steps (APP, EXE, and FOL) during a maximal effort 45° COD task and compare these changes with those during straight running; and (2) compare the GRIs between the EXE and FOL during the COD task. It was hypothesized that: (1) not only the EXE but also the APP and FOL contribute to the directional change needed to achieve the 45° direction change, with the APP and EXE primarily contributing to deceleration, while the FOL contributes to acceleration; and (2) the EXE produces a greater medial GRI than the FOL.

## 2 Materials and methods

### 2.1 Participants

The study included ten Japanese male semi-professional and division 1 collegiate American football players (25.3 ± 3.2 years, 1.73 ± 0.05 m, 77.9 ± 6.1 kg, and 8.6 ± 4.5 years of experience). The participants had no history of major lower limb injury or neuromuscular disorders within the past 3 years. All participants were offensive skill-position players (running backs and wide receivers). Three participants with experience on the Japan national team were classified as Tier 4 (international/elite level), while the remaining seven participants were classified as Tier 3 (highly trained/national level) (18). Power analysis conducted using G*Power (version 3.1.9.2; Heinrich-Heine-Universität Düsseldorf, Düsseldorf, Germany) indicated that a minimum of nine participants would be required to detect a large effect size (*f* = 0.4, corresponding to a partial η^2^ of 0.14) (19) with 95% power at a 5% significance level in a 2 × 3 repeated-measures analysis of variance (ANOVA). Therefore, the sample size of ten participants was deemed sufficient for this study. They provided written informed consent to undergo the experimental procedures. The study was conducted in accordance with the Declaration of Helsinki and approved by the ethics board of the Japan Institute of Sports Sciences (approval number: H26-015).

### 2.2 Procedures

**Figure 1:**
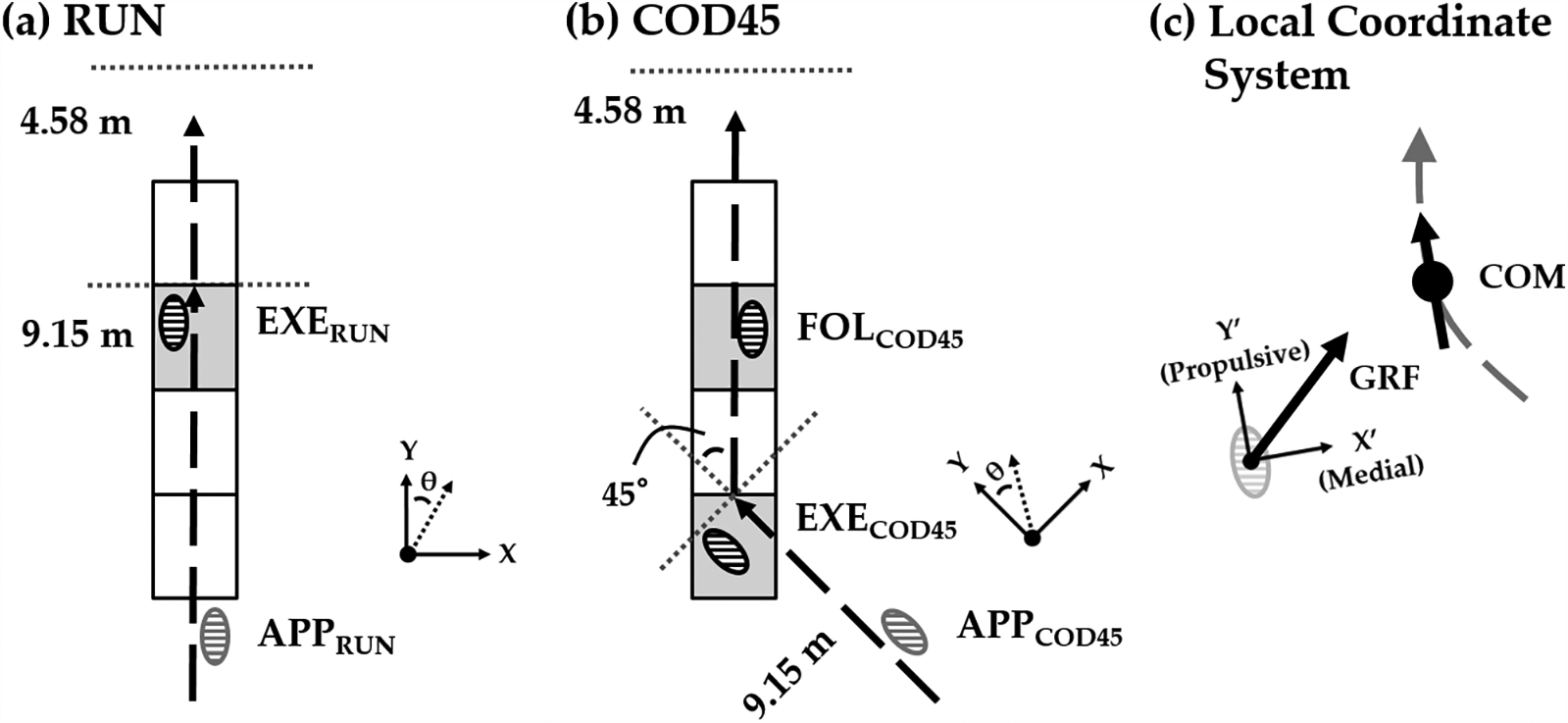
Experimental setup for **(a)** straight running (RUN) and **(b)** 45° change-of-direction (COD45) tasks. APP, approach step; EXE, execution step; FOL, following step. The rectangular grids represent the force platforms. The laboratory reference frame is defined by the X and Y axes, while *θ* represents the direction of the center of mass (COM). **(c)** Definition of the local coordinate system, which is aligned with the COM velocity vector. The gray dashed line represents the path of progression. The local reference frame is defined by the X′ and Y′ axes. The horizontal ground reaction force (GRF) consists of propulsive-braking and medial-lateral components.

Before testing, the participants completed a 15 min standardized warm-up comprised of jogging, dynamic stretching, and sprinting. Then, they performed two tasks on the indoor Tartan track: straight running (RUN) and sidestep cutting to 45° (COD45). For RUN, they ran as fast as possible across a 15-yard (13.73 m) path (Figure 1a). For COD45, they ran as fast as possible across a 10-yard (9.15 m) path, planted their left foot on a force platform at the 10-yard point to change direction for a 45° cut, and then ran an additional 5 yards (4.58 m) to the finish line (Figure 1b). Due to space limitations in the laboratory, the COD45 could only be performed to the right, but since these running paths are common passing routes for offensive skill-position players in American football (20), assessing only one direction is unlikely to affect the experimental outcomes. All participants wore the same model of athletic shoes (LANCAMENTO SL4, Mizuno, Osaka, Japan). Four force platforms were built in the track, covered with the same Tartan mat, and leveled with the track. Running paths were marked on the floor with tape. To ensure consistent placement of the execution step (EXE) on the force platform while minimizing the need for participants to consciously adjust their stride, the starting position was adjusted behind the start line for each participant. Although no specific instructions were provided regarding foot placement for the following step (FOL), participants consistently stepped onto the subsequent force platform across all trials. The testing protocol consisted of four successful trials of each task. The order of the tasks (RUN and COD45) was randomized for each trial to prevent effects related to the testing order.

Three-dimensional (3D) coordinates of the participants' anatomical landmarks were acquired using a 3D optical motion capture system equipped with 20 cameras (250 Hz; Vicon, Oxford, UK). Twenty-eight reﬂective markers were attached to each participant's seventh cervical vertebrae, suprasternal notch, right and left sides of their heads, shoulders, elbows, wrists, hands, anterior superior iliac spines, greater trochanters, heels, toes, medial and lateral knees, and ankles. The COM trajectory was calculated based on body segment parameters (21). The mass of the shoe (0.26 kg) was added to the foot mass for COM calculations (22). Ground reaction force (GRF) data were obtained at 1,000 Hz using force platforms (0.9 m × 0.6 m, type 9287B; Kisler Group, Winterthur, Switzerland).

### 2.3 Data collection

The marker data were low-pass filtered using a fourth-order zero-lag Butterworth filter with a 12 Hz cutoff frequency (10). The GRF data were low-pass filtered using a fourth-order zero-lag Butterworth filter with a 75 Hz cutoff frequency (23), down-sampled to 250 Hz, and synchronized with the kinematic data.

**Figure 2:**
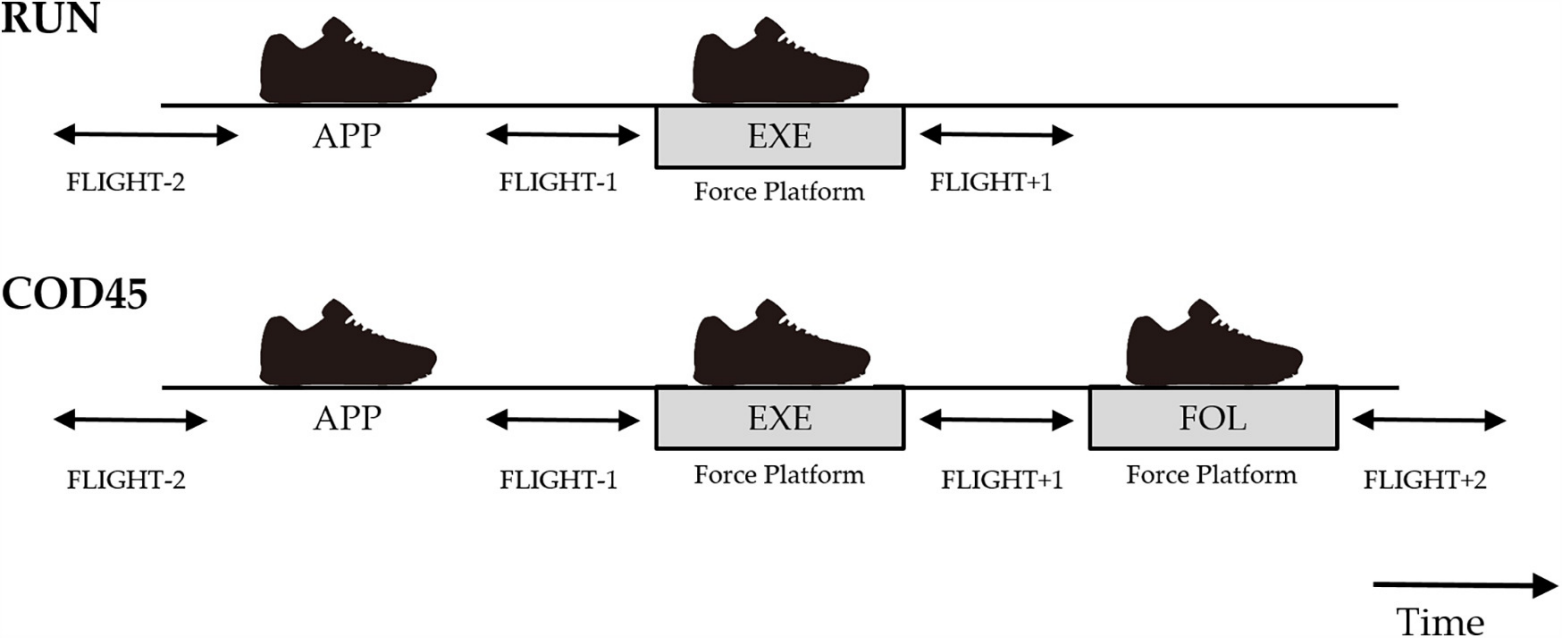
Time course of foot contact and definitions of the flight phases. RUN, straight running; COD45, 45° change of direction; APP, approach step; EXE, execution step; FOL, following step.

The COM speed was defined as the magnitude of the horizontal COM velocity vector, while the COM direction (*θ*) was defined as the angle between the horizontal COM velocity vector and the *Y*-axis of the laboratory reference frame (Figures 1a,b). The execution step was defined as the cut step (EXE), with the preceding step as the approach step (APP), and the subsequent step as the following step (FOL). COM speed and direction were calculated for four flight phases in COD45 and three in RUN (Figure 2). In COD45, these phases corresponded to the flight phases before the APP (FLIGHT-2), between the APP and EXE (FLIGHT-1), between the EXE and FOL (FLIGHT + 1), and after the FOL (FLIGHT + 2). In RUN, these phases corresponded to the flight phases before EXE (FLIGHT-2), between APP and EXE (FLIGHT-1), and after EXE (FLIGHT + 1). Flight phases were identified based on one of the following criteria: (1) the toe and heel markers were more than 0.08 m above the ground, or (2) the vertical GRF was less than 30 N when the foot contacted force platforms. COM speed and direction were calculated by averaging five frames within each flight phase, assuming the COM's horizontal speed and direction remain constant during the flight phase due to the principle of projectile motion.

To assess the components of GRF contributing to changes in the COM speed and direction, horizontal GRFs during EXE in RUN, and EXE and FOL in COD45, were rotated into a local coordinate system (Figure 1c). The local coordinate system was aligned with the COM velocity vector using the three-point finite difference method (14). The rotated GRFs were integrated to determine propulsive, braking, medial, and lateral GRIs, as well as net propulsive and net medial GRIs. The GRIs were normalized by body mass. Theoretically, propulsive and braking GRIs correspond to the increase and decrease in COM velocity during stance, respectively. Similarly, medial and lateral GRIs theoretically represent the change of the COM angle toward and away from the cutting direction, respectively. Foot contact events were defined using a 30 N vertical threshold (10). Due to limitations in the available space within the laboratory, GRFs during FOL and COM speed and direction during FLIGHT + 2 of RUN were not analyzed (Figures 1a, 2). For all dependent variables, the analysis included the average of the fastest three trials during the FLIGHT-1; the slowest trials during the FLIGHT-1 were excluded from the analysis. All numerical calculations were performed using MATLAB 2018b (MathWorks, Inc., Natick, MA, USA).

### 2.4 Statistical analysis

Two-way repeated-measures analysis of variance (ANOVA) was used to determine if differences existed in the COM angle and speed between tasks (RUN and COD45) or flight phases (FLIGHT-2, FLIGHT-1, FLIGHT + 1). When the Mauchly test of sphericity showed heterogeneity of covariance, the more conservative Greenhouse-Geiser test was performed. Significance was set at *α* = .05. *post-hoc* analyses were conducted with paired t-tests to compare the values measured at each flight phase between tasks using Bonferroni correction, *α* < .017 (.05/3). One-way repeated-measures ANOVA was performed to analyze the COM speed and the direction across flight phases within each task. When the effect of the flight phase was determined, *post-hoc* analyses were performed with the Bonferroni test. Paired-sample *t*-tests were also performed to compare GRIs between EXE in RUN and EXE in COD45, as well as between EXE and FOL in COD45. Statistical analyses were performed using IBM SPSS Statistics 19.0 (IBM Corp., Armonk, NY, USA).

## 3 Results

A significant interaction was observed for the COM direction (*F*_(2,18)_ = 870.30, $\eta_{p}^{2}=.990$, *p* < .001) (Table 1). One-way repeated-measures ANOVA revealed a significant main effect of the flight phase in both RUN (*F*_(2, 18)_ = 17.04, $\eta_{p}^{2}=.654$, *p* < .001) and COD45 (*F*_(3, 27)_ = 785.75, $\eta_{p}^{2}=.989$, *p* < .001). Across all flight phases, the COM direction was significantly greater in COD45 than in RUN (*p* < .001), and progressively increased with each flight phase in COD45 (*p* < .001). Individual COM direction data for each participant are presented in the Supplementary Figure.

**Table 1:** COM direction and speed during each flight phase.

Variables	FLIGHT-2	FLIGHT-1	FLIGHT + 1	FLIGHT + 2
Direction (degree)				
RUN	0.84 ± 0.70^a^,^b^	−0.30 ± 0.65^a^,^c^	1.45 ± 0.82^a^	–
COD45	4.38 ± 2.54^b^,^c^,^d^	14.08 ± 2.53^c^,^d^	29.38 ± 2.45^d^	38.43 ± 1.33
Speed (m/s)				
RUN	6.88 ± 0.03^a^,^b^,^c^	7.21 ± 0.30^a^,^c^	7.51 ± 0.32^a^	–
COD45	6.43 ± 0.26^b^,^d^	6.66 ± 0.29	6.55 ± 0.35^d^	6.68 ± 0.34

COM, center of mass; RUN, straight running; COD45, 45° change of direction.

^a^ Significant difference compared to COD45 (*p* < .05).

^b^ Significant difference compared to Flight-1 (*p* < .05).

^c^ Significant difference compared to Flight + 1 (*p* < .05).

^d^ Significant difference compared to Flight + 2 (*p* < .05).

In the comparison of EXE between RUN and COD45, the net medial and medial GRIs were greater in COD45 than in RUN (*d* = 15.048, *p* < .001 and *d* = 15.400, *p* < .001, respectively). Within COD45, both EXE and FOL generated predominantly medial GRFs, with negligible lateral GRIs. Comparisons between EXE and FOL in COD45 revealed that the net medial and medial GRIs were significantly greater during EXE than during FOL (*d* = 3.282, *p* < .001 and *d* = 3.294, *p* < .001, respectively). The medial GRI during EXE was greater than that in FOL (*d* = 2.243, *p* < .001), whereas the lateral GRI did not differ significantly (*d* = 0.501, *p* = .220).

A significant interaction was observed for the COM speed (*F*_(2,18)_ = 105.69, $\eta_{p}^{2}=.922$, *p* < .001) (Table 1). One-way repeated-measures ANOVA revealed a significant main effect of the flight phase in both RUN (*F*_(2, 18)_ = 491.74, $\eta_{p}^{2}=.982$, *p* < .001) and COD45 (*F*_(1.430, 12.873)_ = 11.76, $\eta_{p}^{2}=.567$, *p* = .002). The COM speed was consistently lower in COD45 than that in RUN across all flight phases (*p* < .05). In RUN, the COM speed increased progressively across all flight phases (*p* < .001). In COD45, the COM speed increased significantly from FLIGHT-2 to FLIGHT-1 (*p* = .001), remained unchanged between FLIGHT-1 and FLIGHT + 1 (*p* = .053), and increased again from FLIGHT + 1 to FLIGHT + 2 (*p* = .003). Individual COM speed data for each participant are also provided in the Supplementary Figure.

In the comparison of EXE between RUN and COD45, the net propulsive GRI was greater in RUN than in COD45 (*d* = 1.078, *p* = .027), while the propulsive GRI did not differ significantly (*p* = .463, *d* = 0.243). The braking GRI was significantly greater during EXE in COD45 compared to RUN (*p* < .001, *d* = 3.321). Within COD45, the net propulsive GRI was greater during FOL than during EXE. In contrast, both braking and propulsive GRIs were smaller during FOL compared to EXE (*p* = .003, *d* = 1.267 and *p* = .002, *d* = −1.331, respectively).

Stance time was longer during EXE in COD45 than in RUN (*p* < .001, *d* = 2.701), but did not differ between EXE and FOL within COD45 (*p* = .623, *d* = 1.590) (Table 2).

**Table 2:** Variables obtained from force platforms during the execution (EXE) and following (FOL) steps.

Variables	Task	EXE	FOL
Stance time (s)	RUN	0.131 ± 0.008^a^	–
	COD45	0.152 ± 0.008	0.149 ± 0.013
Relative GRI (Ns/kg)			
Net Medial	RUN	0.13 ± 0.09^a^	–
	COD45	1.75 ± 0.12^b^	1.02 ± 0.29
Medial	RUN	0.16 ± 0.08^a^	–
	COD45	1.75 ± 0.12^b^	1.02 ± 0.29
Lateral	RUN	0.03 ± 0.02^a^	–
	COD45	0.00 ± 0.00	−0.00 ± 0.00
Net Propulsive	RUN	0.29 ± 0.04^a^	–
	COD45	0.02 ± 0.10^b^	0.12 ± 0.10
Propulsive	RUN	0.39 ± 0.00	–
	COD45	0.38 ± 0.05^b^	0.29 ± 0.04
Braking	RUN	0.10 ± 0.02^a^	–
	COD45	0.36 ± 0.08^b^	0.17 ± 0.09

EXE, execution step; FOL, following step; GRI, ground reaction impulse; RUN, straight running; COD45, 45° change of direction.

^a^ Significant difference compared to COD45 (*p* < .05).

^b^ Significant difference compared to FOL (*p* < .05).

## 4 Discussion

This is the first study to focus on changes in COM direction and speed over three steps (APP, EXE, and FOL) during COD45 performed with maximal effort. Partial support for the hypotheses was found, demonstrating that the APP, EXE, and FOL all contributed to the directional change. However, even with the combined contributions of these three steps, the athletes did not achieve the full 45° change of direction. Contrary to our initial expectations, the APP and EXE did not contribute to deceleration; instead, COM speed increased during APP and remained relatively unchanged during EXE, while the FOL contributed to acceleration as hypothesized. These results confirm that completing a 45° COD task with maximal effort requires a multi-step strategy that incorporates both directional changes and acceleration/speed maintenance across multiple steps.

The EXE produced a longer stance time for the COD45 task than that for the RUN task, which contributes to a greater GRI (10) and a greater direction change (24). Our study revealed that the EXE produced a greater medial GRI compared to FOL. Nevertheless, the angle change during EXE (between FLIGHT-1 and FLIGHT + 1) was only one-third (15.30°) of that required for the task (45°), even though EXE is defined as the “plant foot” (25, 26). This result is supported by Vanrenterghem et al. (11), who revealed that the directional change during EXE in the 45° COD task decreased with higher approach speeds (from 34.9° at 2 m/s–17.5° at 5 m/s). Considering that the direction of the COM velocity vector is calculated by the fore-aft and medial-lateral components, one can appreciate that as the approaching speed increases, more of the medial component of the COM velocity is required to change the same angle. If a player cannot produce enough medial COM velocity during EXE, then contribution of the FOL is required. This finding is consistent with the angle-velocity trade-off model (27), which highlights that higher approach velocities constrain the achievable cutting angle. Notably, the approach velocity in our study was considerably high (6.66 m/s), supporting the interpretation that the limited directional change during EXE is a natural consequence of maximizing entry speed during COD.

Our findings revealed that the FOL also contributed to the COD to some extent (9.05°; the direction change between FLIGHT + 1 and FLIGHT + 2), producing less medial GRF impulse compared with the EXE, although the stance time during the FOL was similar to that during the EXE. This finding quantitatively supports the concept proposed by Dos'Santos et al. (12), who suggested that crossover cuts are typically performed following the main execution sidestep as part of a multi-step action. Even though participants did not achieve a 45° COD after the FOL (reaching only 38.43°), this strongly suggests that the directional change during the FOL was performed with maximal effort. While various offensive and defensive agility techniques exist in invasion sports, the primary techniques involving directional changes with speed maintenance/acceleration are the sidestep cut and crossover-step cut (28, 29). The sidestep cut occurs as an athlete plants his/her foot opposite to the new direction, whereas the crossover-step cut occurs as the athlete plants his/her foot on the same side of the new direction, and then crosses the opposite leg (17). Previous studies have conducted sidestep and crossover-step cut trials, and showed that the COM direction change was greater during the sidestep cut than during the crossover-step cut (30–32). Applying these maneuvers to the current study, the EXE acts as the sidestep cut, the FOL (and potentially the APP, with a 9.70° directional change) acts as the crossover-step cut, and the EXE has the capacity to produce a greater direction change compared with the FOL. Therefore, our results suggest that the sidestep and crossover-step maneuvers were used continuously by the athletes, not separately, to perform a COD with maximal effort.

In invasion sports, an offensive player generally needs to minimize the lateral displacement of the COM before EXE, and then change to the final running direction to prevent anticipation of this movement by a defensive player (33, 34). However, participants initiated the COD maneuver prior to EXE (14.08°), with APP contributing 9.70° to the direction change (difference between FLIGHT-2 and FLIGHT-1). This finding is consistent with those reported in previous studies. Havens and Sigward (10) confirmed that an offensive player produced lateral GRF during APP. Furthermore, Fujii et al. (35) revealed that a defensive player anticipated the future direction of an offensive player before EXE. These anticipatory adjustments during the APP, which have been suggested to reduce stress on the EXE leg and alter whole-body posture (36), were also necessary to accomplish the task. The combined angle change during EXE and FOL (24.35°) only achieved approximately half of the required 45°, highlighting the need for these pre-EXE adjustments. Although the task in this study was intentionally designed to replicate pre-planned offensive passing routes in American football, real-game situations often require reactive adjustments to prevent defenders from anticipating the movement. Thus, it should be noted that the pre-planned nature of the task may not fully replicate the movement strategies required during actual field play. In particular, pre-planned settings may lead to a more rounded movement pattern prior to COD (37), representing a limitation of the experimental design.

Regarding the change in speed during COD, the EXE has been considered to play a decelerating role (17), contrasting with an accelerating phase during EXE in RUN (38, 39). However, our study found a similar COM speed between FLIGHT-1 (6.66 m/s) and FLIGHT + 1 (6.55 m/s). While the braking GRI during EXE in COD45 (0.36 Ns/kg) was significantly greater than that during EXE in RUN (0.10 Ns/kg), our results also showed that a comparable propulsive GRI was generated during EXE in both COD45 (0.38 Ns/kg) and RUN (0.39 Ns/kg). Consequently, the net propulsive GRI during EXE in COD45 was almost zero (0.02 ± 0.10 Ns/kg), the primary determinant of COM speed changes (38, 39), and consequently COM speed remained relatively unchanged before and after EXE (i.e., between FLIGHT-1 and FLIGHT + 1). This suggests that while EXE produces a large braking GRI, it does not necessarily lead to a net decrease in COM speed during COD tasks.

During FOL in the COD45 task, the COM speed significantly increased by 0.13 m/s (the difference between FLIGHT + 1 and FLIGHT + 2). This is because the propulsive GRI during FOL was smaller than that during EXE, yet the COM speed increased by a much smaller braking GRI than that during EXE. Previous studies have considered that the FOL acts as the re-acceleration that is required to gain separation from an opponent (2, 17); however, those studies have not quantified the amount of acceleration. Our study confirmed that the FOL increases the COM speed while producing a less propulsive GRI than that for the EXE.

Our results provide additional insights into the multi-step strategy during a COD task, especially for the difference between maximal and submaximal effort. Many studies have controlled the COM speed while keeping the experimental condition constant, and have measured the joint kinematics and kinetics (8, 11, 16, 30, 31, 36, 40). In this experimental setting, the participants may possibly decelerate during APP (8, 16) to prevent local stress of the lower limb in EXE and re-accelerate during FOL instead (15). However, athletes usually perform a COD task within a short distance with acceleration, and then attempt to accelerate again in the new direction, to produce as much propulsive and medial GRIs at every single step as possible (41). In the current study, the COM speed increased by 0.23 m/s during APP in COD45 (from 6.43 m/s during FLIGHT-2–6.66 m/s during FLIGHT-1). This increased amount tended to be smaller than that in RUN (0.33 m/s); however, we confirmed that APP played a role in accelerating the COM speed with maximal effort COD. Our result supports the finding from a previous COD study that compared the COM speed with maximal effort before and after APP (an increase of 0.12 m/s) (10). Therefore, the strategies to complete the COD task with multiple steps are different from the maximal and submaximal effort. Additionally, this performance strategy in sports situations may cause lower limb injuries. A previous study has reported that most non-contact anterior cruciate ligament (ACL) injuries in rugby union occur during sidestep cuts by ball carriers (42). In response, technique modification training for COD maneuvers has emphasized deceleration during APP and optimizing of EXE mechanics to reduce injury risk (43, 44). However, our findings show that FOL also contributes to directional change through medial impulse production. Therefore, training strategies should promote role-sharing across steps, with FOL assisting directional change to reduce the mechanical demands on APP and EXE, offering a broader perspective for injury prevention.

This study had some limitations. The RUN and COD45 were performed on a synthetic track rather than on artificial or natural turf with cleats, and differences in the coefficient of friction may have altered the force application during the COD task (45), which in turn could have affected speed and agility performance (46, 47). Although participants were offensive skill-position players accustomed to cutting in both directions, analyzing only right-sided cuts may have introduced bias related to limb dominance or asymmetry. Additionally, while the sample size was sufficient to detect large effects based on power analysis, the relatively small number of participants may limit the generalizability of the findings. Furthermore, interpretation of these results may be limited because the COD strategy would differ depending on the task (approach distance or directed angle), instruction, and the player's skill level (10, 27). Despite these limitations, the findings provide valuable insights for practitioners to understand the performance of COD with maximal effort in sports.

## 5 Conclusion

In a maximal effort 45° COD task, the EXE produced a greater medial GRF impulse than the FOL, while COM speed remained relatively unchanged. However, the directional change during the EXE (15.30°) reached only one-third of the required 45°. The APP and FOL also contributed to the directional change, while simultaneously increasing COM speed. These findings highlight that completing a 45° COD task with maximal effort requires a multi-step strategy, with continuous use of sidestep and crossover-step maneuvers, rather than relying solely on the EXE.

## Data Availability

The raw data supporting the conclusions of this article will be made available by the authors, without undue reservation.

## Supplementary material

Supplementary Figure Individual plots of center of mass (COM) direction and speed during each flight phase. Each line represents data from a single participant. RUN, straight running; COD45, 45° change of direction.
