# Supplementary figures and images for: Center of mass direction and speed during a 45-degree change of direction task performed with maximal effort
Source: Front Sports Act Living. 2025 Jun 5;7:1576614. doi: 10.3389/fspor.2025.1576614 (PMC12179697; doi:10.3389/fspor.2025.1576614)

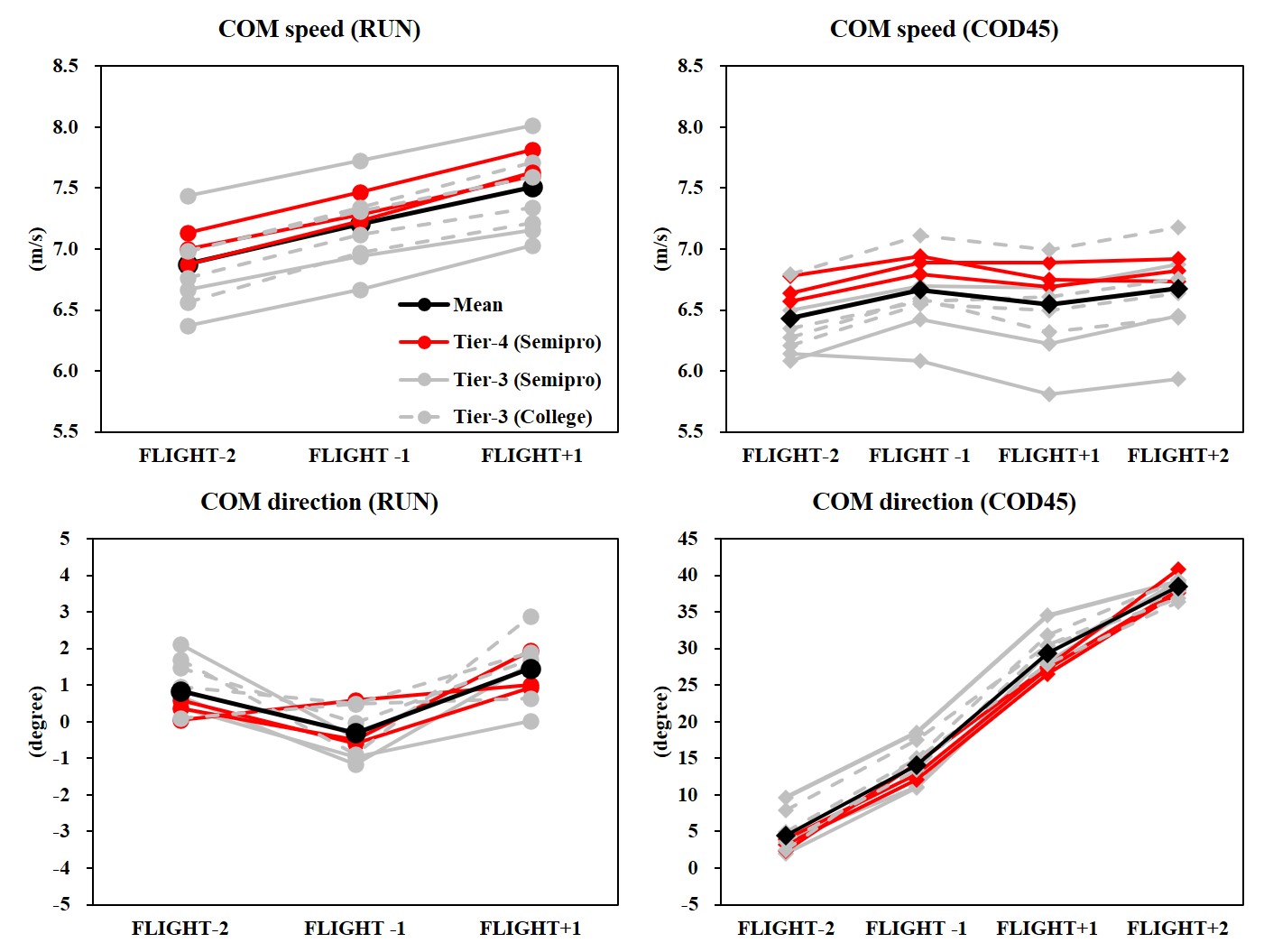

Supplement: Supplementary Figure — Individual plots of center of mass (COM) direction and speed during each flight phase. Each line represents data from a single participant. RUN, straight running; COD45, 45° change of direction. [file Image1.jpg]
